# Hydrogel Microdomain Encapsulation of Stable Functionalized Silver Nanoparticles for SERS pH and Urea Sensing

**DOI:** 10.3390/s19163521

**Published:** 2019-08-12

**Authors:** Alexander Quinn, Yil-Hwan You, Michael J. McShane

**Affiliations:** 1 Department of Chemical Engineering, Texas A&M University, College Station, TX 77843, USA; 2 Department of Materials Science and Engineering, Texas A&M University, College Station, TX 77843, USA; 3 Department of Biomedical Engineering, Texas A&M University, College Station, TX 77843, USA; 4 Center for Remote Health Technologies and Systems, Texas A&M Engineering Experiment Station, College Station, TX 77843, USA

**Keywords:** SERS, implantable sensors, urease, urea, minimally-invasive sensors, pH biosensors

## Abstract

Conceptual and commercial examples of implantable sensors have been limited to a relatively small number of target analytes, with a strong focus on glucose monitoring. Recently, surface-enhanced Raman spectroscopy (SERS) pH sensors were demonstrated to track acid-producing enzymatic reactions targeting specific analytes. We show here that SERS pH tracking in the basic regime is also possible, and can be used to monitor urea concentration. To accomplish this, we developed a hydrogel consisting of polyelectrolyte multilayer microcapsules containing a SERS-sensitive pH reporter (4-mercapopyridine capped silver nanoparticles modified with bovine serum albumin). This pH sensing material exhibited a sensitive Raman scattering response to a wide range of pH from 6.5–9.7. By incorporating urease into the hydrogel matrix, the new sensor was capable of distinguishing urea concentrations of 0, 0.1, 1, and 10 mM. We also found that bovine serum albumin (BSA) prevented severe aggregation of the nanoparticle-based pH sensor, which improved sensing range and sensitivity. Furthermore, BSA safeguarded the pH sensor during the encapsulation procedure. Together, the combination of materials represents a novel approach to enabling optical sensing of reactions that generate pH changes in the basic range.

## 1. Introduction

Minimally-invasive implantable sensors offer exciting possibilities to improve patient care and diagnostics [1] with continuous time-resolved analyte concentration measurements [2], local probing, remote monitoring [3,4], and better patient acceptance [5]. As an example, the Senseonics Eversense^®^ glucose sensor recently debuted as the first fully-implantable commercial sensor to receive FDA approval [6]. Human-implantable sensors for other analytes, though, do not exist and require new sensing chemistries.

Enzymes are popular sensor components for their analyte-selective reactions and easy integration [7]. Enzymatic reactions may be tracked by measuring reactant consumption, product accumulation, or possibly accompanying environmental changes. Solution pH change, for instance, results from many enzymatic reactions and can be measured optically via chemicals sensitive to pH change. Transduction of pH in optical biosensors is often accomplished with colorimetric dyes or fluorescent indicators [8,9,10,11,12,13], which are sensitive but tend to lose function over time. An emerging and promising alternative technique that offers improved stability is surface enhanced Raman spectroscopy (SERS), which is made possible by the strong amplification of the weak Raman scattered light using plasmonic materials. Preparation of an implantable SERS sensor is possible if the plasmonic substrates are properly encapsulated in a biocompatible matrix. The materials aspect of this was recently demonstrated, wherein pH change induced by glucose oxidation via glucose oxidase enzyme was measured with a 4-mercaptobenzoic acid (MBA) SERS pH reporter [14,15].

In principle, using the same SERS pH reporter, the glucose oxidase enzyme could be replaced with another acid-producing enzyme to create a new sensor, offering an avenue to expand the sensing platform to other targets in a “mix-and-match” approach. However, MBA only responds to pH changes in the acidic range. Consequently, the SERS reporter is a limitation to the possibilities of the system. A SERS reporter that functions in the basic pH region would offer additional capabilities, allowing development of sensors that track enzymatic reactions which drive pH increases, such as creatinine catalysis by creatinine iminohydrolase [16], or urea by urease [8,17,18]. In general, ammonia-producing enzymatic reactions can alkalize solution by NH_3_ +2H_2_O ↔ NH_4_^+^ + OH^−^ + H_2_O [19]. These reactions may be tracked through increases in pH, if acid is not concurrently produced.

Urea sensing provides a relevant application example. Implantable urea sensors could replace single-point blood urea nitrogen or urea clearance tests to continuously monitor renal and hepatic function. Currently such measurements are made infrequently (on the order of three-month intervals), even for patients with severe kidney or liver conditions; providing frequent/constant monitoring would enable patients to more dynamically adjust to changing health conditions. Urea hydrolysis by urease, (NH_2_)_2_CO + 3H_2_O → 2NH_4_^+^ + HCO_3_^−^ + OH^−^ [18], produces hydroxide base which can be probed by a pH sensor. This method is used in various research sensors, many of which are studied for on-line dialysis measurement [17,20,21,22,23,24,25,26,27,28,29]. Inspired by the potential for in vivo sensors, a fluorescence-based urea sensing approach was also demonstrated: pH-sensitive fluorescent dye, urease, and reference dye were encapsulated in polyelectrolyte multilayer microcapsules to measure urea by tracking pH [8]. System pH changes, though, were only discerned in deionized water and not in buffer, making this sensor infeasible to use in body fluids. The lack of pH sensitivity in buffered solution may be attributed to solution measurement volume, low urease activity, and length of measurement, which we believe may be overcome with our SERS-based sensor.

SERS has been demonstrated as a technique applicable to in vivo measurement. However, the substrates employed required surgical implantation and elicited significant immune response [30]. To address issues related to implantation and sensing through skin, hydrogel-based pH, glucose, and microRNA-17 sensors using encapsulated SERS reporters have been developed [14,15,31]. Multiple aspects of SERS sensors are desirable over fluorescent ones. SERS reporter molecules have ‘fingerprint’ spectra that provide multiple peaks for molecule identification/confirmation and the potential for signal multiplexing [32]. A key aspect of selective signal amplification arises from the fact that enhancement is highly localized to the substrate surface: this restricts sensing to near the SERS substrate and dramatically increases scattering signal levels above background Raman scattering and fluorescence. Certain nanostructures form SERS hot spots which further enhance scattering intensity [33]. SERS reporter molecules are often simple compared to conjugated dyes and are not subject to photo-bleaching, lending superior stability to the chemo-optical transduction approach. Lastly, it is important to note that SERS substrates can be tuned to respond to near-infrared excitation sources which can penetrate skin tissues [34,35,36,37]. 

For urea sensing, the pH sensitive ligand must report at and above the pH of body fluids (pH > 7.4). A candidate for this is 4-mercaptopyridine (4-MPy); gold-nanoparticles functionalized with 4-MPy and protected with bovine serum albumin (BSA) responded to at least pH 9 [38]. Furthermore, the 4-mercaptopyridine molecule is a well-studied SERS ligand and pH reporter [39,40,41,42,43,44,45,46]. Here, we chose to use 4-MPy as the basis for our sensor, with the intention of developing an appropriate functionalization and hydrogel encapsulation approach to enable implantation.

For implantable sensors, the SERS-active nanoparticles and the urease enzyme must be immobilized to prevent dispersion into body fluids. One strategy is to encapsulate the SERS sensors in microcapsules, immobilize the microcapsules in a hydrogel, and then conjugate enzyme to the hydrogel. Using this strategy, system modularity can be achieved by immobilizing microcapsules with different sensors and functions [47]. Microcapsules can be simply fabricated via layer-by-layer polyelectrolyte deposition, readily accept a variety of cargo, and have tunable size and permeability [8,10,48,49,50]. Polyelectrolyte microcapsules consisting of poly (diallyldimethylammonium chloride) (PDADMAC) and polystyrene sulfonate (PSS) polyelectrolytes were chosen for this study because of their relatively high permeability and proven ability to encapsulate nanoparticle chemistry [15]. The biocompatible matrix immobilizes the sensor contents but also serves to resist foreign-body response upon implantation [14,15,51,52]. Alginate was chosen as a model hydrogel system for this work because of its easy synthesis, mechanical flexibility, easy integration with the microcapsules/urease, and low scattering contribution to Raman signal [15,51]. Alginate hydrogel, BSA-modified AgNPs, and PDADMAC have shown limited biocompatibility [53,54,55] whereas PSS coated on gold nanorods exhibited cytotoxicity [56]. These materials, though, are embedded in the hydrogel, whose exposed surface determines the system biocompatibility [57]. Thus, tuning the hydrogel characteristics is key to addressing biocompatibility, which is not explored in this work.

**Figure 1:**
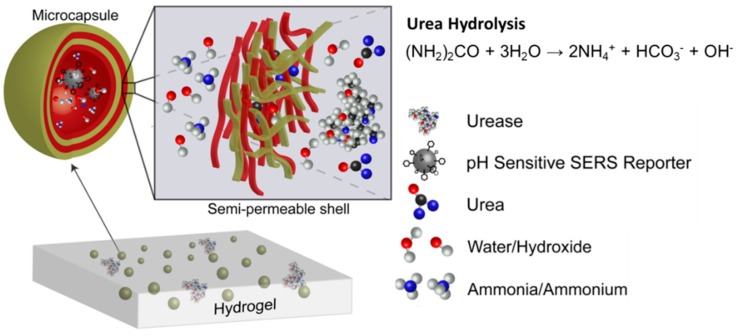
Microcapsules containing surface-enhanced Raman spectroscopy (SERS) pH reporter, immobilized in an alginate hydrogel.

In this study, silver nanoparticles functionalized with 4-MPy (MPy-AgNP) were tested as a pH sensor. MPy-AgNP was subsequently modified with BSA to improve the pH response in the basic region [38]. Silver nanoparticles were used because of their easy synthesis and high SERS amplification relative to gold nanoparticles. The BSA-modified MPy-AgNPs were then encapsulated in polyelectrolyte multilayer capsules and suspended in alginate hydrogel with and without urease, to create a working pH and urea sensor respectively. The SERS spectra of the colloidal and hydrogel systems are compared. In the urea sensor system (Figure 1), urea is hydrolyzed to yield an increased solution pH, which is then directly reflected in the 4-MPy SERS and correlated to urea concentration.

## 2. Materials and Methods

### 2.1. Chemicals

Hydroxylamine hydrochloride, silver nitrate, PSS (avg Mw = 70,000), PDADMAC (avg Mw = 100–200 kDa), urea, 4-mercaptopyridine, bovine serum albumin, trizma maleate, Dulbecco’s phosphate-buffered saline, sodium carbonate, sodium bicarbonate, ethane sulfonic acid (MES) sodium salt, 1-ethyl-3-(3-dimethylaminopropyl)-carbodiimide hydrochloride (EDC), and alginic acid sodium salt (from brown algae, ∼250 cps for 2% solution at 25 °C) were obtained from Sigma-Aldrich (St. Louis, MO, USA). Calcium chloride was obtained from Macron Fine Chemicals (Center Valley, PA, USA). Sulfo N-hydroxysuccinimide (NHS) was obtained from Thermo Fisher Scientific (Waltham, MA, USA).

### 2.2. Silver Nanoparticle Synthesis

Hydroxylamine hydrochloride coated silver nanoparticles (AgNPs) were synthesized by reduction of silver salt [58]. NaOH (11.9 mg) and 10.4 mg of hydroxylamine hydrochloride were stirred in 90 mL of deionized (DI)water. AgNO_3_ (16.9 mg) in 10 mL of DI water was added dropwise and stirred for 15 min resulting in AgNPs with an average concentration of 0.7 nM and 30–40 nm diameter, as estimated by UV-vis [59]. 

### 2.3. AgNP Monolayer Modification

MPy-AgNP and BSA-modified MPy-AgNP were prepared in solution. Over 30 s, 0.5 mM 4-MPy solution was added to 10 mL of stirring AgNPs and the solution stirred for an extra 15 min to achieve MPy-AgNP. For the BSA-modified MPy-AgNP, 4 w/v% BSA was added during the 15 min stirring period. The 4-MPy molecule to total AgNP surface area ratio was kept constant at 3.5 molecules/nm^2^ by adding the respective amount of 0.5 mM 4-MPy solution. For the BSA-modified MPy-AgNP, the BSA molecule to AgNP surface area ratio was kept at 4.1 molecules/nm^2^ (66.5 kDa BSA) by adding the respective amount of 4 w/v% BSA. AgNP surface area was calculated by assuming spherical particle geometry using the mean diameter and considering the volume and concentration of each sample. For pH measurement, MPy-AgNP or BSA-modified MPy-AgNP samples were centrifuged once at 4000 g for 10 min and the DI water replaced with buffer.

### 2.4. Encapsulation of BSA-Modified MPy-AgNPs in Microcapsules

Polyelectrolyte multilayer microcapsules consisting of 10 PDADMAC/PSS layers and containing BSA-modified MPy-AgNPs were fabricated using an established method [47], with one modification: BSA-modified MPy-AgNPs (400 µL concentrated from 2 mL) was used instead of MBA-AuNPs. For measurement, samples were washed three times, suspended in pH 7.4 PBS 1× buffer, and equilibrated for 30 min before SERS. Samples used for hydrogel fabrication were unwashed.

### 2.5. Fabrication of pH Sensing Hydrogel

Microcapsules containing BSA-modified MPy-AgNPs were immobilized in hydrogel using a similar procedure as used to immobilize microcapsules in alginate [47,51]. Either 150 µL (1×), 600 µL (4×) or 1.8 mL (12×) of microcapsule dispersion was concentrated to 125 µL, mixed with 25 µL of 10 mM calcium chloride solution, added to 250 µL of 3 w/v% alginate solution, and mixed by pipette with 125 µL of 0.5 M MES at pH 5.8. After mixing, the mixture was quickly injected between two glass slides separated by a 750 µm thick Teflon spacer. After gelation, the hydrogel sample was stored in 10 mM Tris-maleate with 10 mM CaCl_2_ at pH 7. For pH measurement, 2 mm diameter samples were excised with a biopsy punch, washed three times in 10 mM Tris-maleate with 10 mM CaCl_2_ at pH of interest, and equilibrated for at least 30 min before SERS measurement.

### 2.6. Fabrication of Urea Sensing Hydrogel

Urease and BSA-modified MPy-AgNPs were immobilized using a procedure similar to that used for microcapsules and glucose oxidase in alginate [47,51]. Briefly, urease (5 mg) was dissolved in 250 µL of alginate precursor overnight. Microcapsule dispersion (1.8 mL, 12×) was concentrated to 125 µL and mixed with 25 µL of 10 mM calcium chloride solution and 250 µL of 3 w/v% alginate solution. The mixture was mixed by pipette with 125 µL of 0.5 M MES at pH 5.8 and then quickly injected between two glass slides separated by a 750 µm thick Teflon spacer. After gelation, urease was conjugated to the hydrogel by storing the hydrogel overnight in 10 mM Tris-maleate buffer at pH 7 containing 10 mM CaCl_2_, 7 mg EDC, and 20 mg of NHS. Afterwards, the hydrogel was washed 3 times with the same buffer but without EDC and NHS to remove excess reactant. Hydrogel samples 2 mm in diameter were excised with a biopsy punch and washed 3 times in 10 mM Tris-maleate with 10 mM CaCl_2_ at pH 7. For urea sensing measurements, each disc was placed in a well of a 384-well plate. 30 µL of 10 mM Tris-maleate buffer with 10 mM CaCl_2_ and urea concentration of interest was added to each disc. SERS measurements were immediately taken at regular time intervals.

**Scheme 1:**
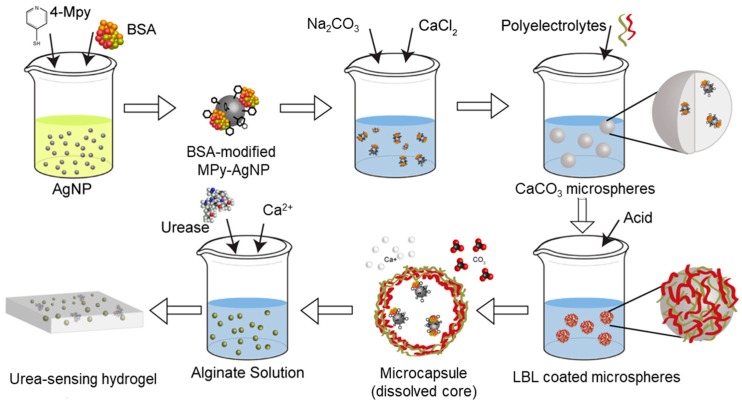
Synthesis of bovine serum albumin (BSA)-modified silver nanoparticles capped with 4-mercaptopyridine (AgNP-MPy), microcapsules containing BSA-modified MPy-AgNPs, and the urea sensing hydrogel.

Scheme 1 outlines the synthesis of BSA-modified MPy-AgNP, the intermediate CaCO_3_ microspheres, microcapsules containing BSA-modified MPy-AgNPs, and the urea sensing hydrogel. First, BSA-modified MPy-AgNPs were made by addition of 4-MPy and BSA into the nanoparticle solution, similar to as reported but using AgNPs instead of gold nanoparticles [38]. MPy-AgNP (not shown in the scheme) was made with the same procedure but without BSA addition. The BSA-modified MPy-AgNP pH sensor was co-precipitated into CaCO_3_ microspheres. A layer-by-layer procedure was used to coat microspheres with polyelectrolyte multilayers. Subsequently the CaCO_3_ core was dissolved with acid resulting in microcapsules containing BSA-modified MPy-AgNPs, hereafter referred to as microcapsules. Microcapsules and urease were mixed with alginate solution which was crosslinked with Ca^2+^, yielding a gel. 

### 2.7. UV-vis Measurements

A Tecan Infinite M200 Pro plate reader and transparent Greiner-Bio Cellstar flat-bottom 96 well plates were used to collect MPy-AgNP and BSA-modified MPy-AgNP extinction spectra. Samples (10 µL) were diluted with 90 µL of buffer. Spectra were collected between 300–800 nm with 1 nm resolution.

### 2.8. SERS

SERS measurements were acquired with a DXR confocal microscope system (Thermo Scientific, Waltham, MA, USA). A 532 nm, 2 mW green laser was used as the excitation source and scattered light collected with a 10× objective lens. The laser was focused on either the surface of the solution or of the excised hydrogel discs. The integration time for a single spectrum was three seconds and five spectra were averaged for each measurement. Background signals and baseline offsets were subtracted using Crystal Sleuth software (Bob Downs, and Tom Laestch of the RRUFF™ Project).

## 3. Results and Discussion

### 3.1. Sensor Synthesis

**Figure 2:**
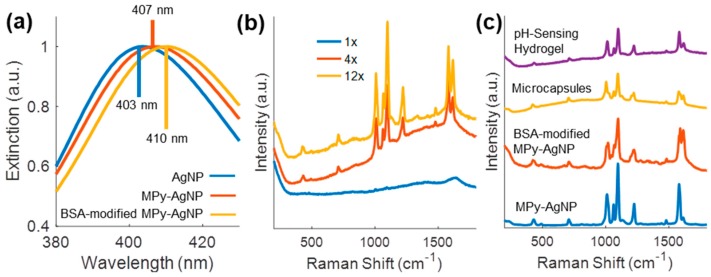
(**a**) Normalized ultraviolet-visible (UV-Vis) spectra of AgNPs, MPy-AgNPs, and BSA-modified MPy-AgNPs in deionized water. (**b**) Raw surface enhanced Raman spectroscopy (SERS) of pH sensing hydrogel with varied microcapsule loading (Tris-maleate, 10 mM CaCl_2_, pH 7). (**c**) Raw SERS of MPy-AgNPs, BSA-modified MPy-AgNPs, microcapsules, and pH sensing hydrogel (Tris-maleate, 10 mM CaCl_2_, pH 7).

MPy-AgNPs, BSA-modified MPy-AgNPs, microcapsules, and the pH/urea-sensing hydrogels were successfully synthesized. The extinction peak red-shift in UV-Vis data from AgNPs to MPy-AgNPs to BSA-modified MPy-AgNPs suggested increased surface modification (Figure 2a). Visually, the MPy-AgNP dispersion was darker, indicating larger aggregates compared to BSA-modified MPy-AgNPs and uncapped AgNPs (Figure S1), as expected [38]. BSA may have prevented excessive aggregation of BSA-modified MPy-AgNPs during the co-precipitation step of the microcapsule fabrication. During co-precipitation, injection of AgNP or MPy-AgNP colloid into the Na_2_CO_3_ solution darkened the mixture to a gray hue, indicating aggregation. In contrast, when BSA-modified MPy-AgNPs were injected into the Na_2_CO_3_ solution, the mixture adopted the yellow color of BSA-modified MPy-AgNPs (Figure S2). During all subsequent fabrication steps (Scheme 1), the yellow color was retained, including in the final hydrogel (Figure S3). The consistent yellow color of the samples throughout processing was notable because: (1) acid used to dissolve the microcapsule cores induced aggregation of BSA-modified MPy-AgNPs and (2) Ca^2+^ used to co-precipitate carbonate microspheres also induced aggregation (further discussed in Section 3.3).

As the compositional complexity of the sensor increased from the BSA-modified MPy-AgNPs, to the microcapsules, to the pH sensing hydrogel, the Raman background increased. Most notably, the background scattering of alginate in the pH sensing hydrogel lowered the SERS signal-to-noise ratio. However, adequate signal-to-noise ratio of 4-MPy SERS was achieved by increasing the microcapsule concentration in the pH sensing hydrogel by 4× and 12× (Figure 2b). The retained 4-MPy fingerprint among all forms of the sensor indicated the BSA-modified MPy-AgNP sensing chemistry was successfully encapsulated and immobilized (Figure 2c). The urea sensing hydrogel SERS spectrum was similar to the pH sensing one, and is not shown.

### 3.2. BSA Corona Improves MPy-AgNP pH Response

**Figure 3:**
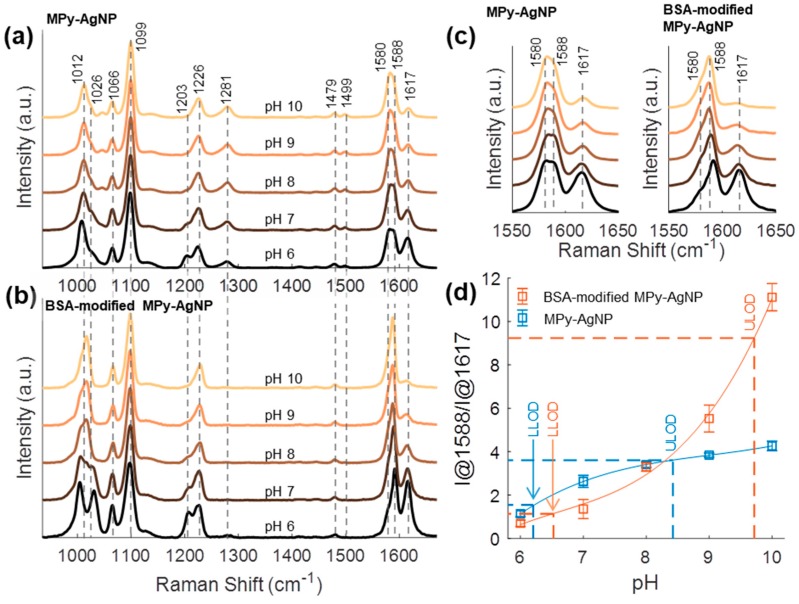
Normalized SERS spectra of (**a**) MPy-AgNPs and (**b**) BSA-modified MPy-AgNPs at pH 6–10. (**c**) Normalized SERS spectra zoomed into region of pH sensing bands. (**d**) pH calibration curves for MPy-AgNPs and BSA-modified MPy-AgNPs. Error bars are one standard deviation (N = 5 for BSA-modified MPy-AgNPs, N = 3 for MPy-AgNPs). Buffer for all samples was 1× PBS.

BSA-modified MPy-AgNP and MPy-AgNP were compared for response to pH to demonstrate that BSA-stabilization is applicable to the AgNP substrate. Nanoparticle-nanoparticle and nanoparticle-MPy-nanoparticle interactions are known to alter the 4-MPy SERS response, including at pH sensitive bands. Such interactions can be prevented with species that sterically or electrostatically prevent nanoparticles from aggregating, for example by adding BSA to an MPy-gold-nanoparticle system [38,45]. In one of these studies, it was found that BSA-modified gold nanoparticles capped with 4-MPy had a single band at 1574 cm^−1^ whereas the BSA-lacking counterpart had bands at 1574, 1582, and 1607 cm^−1^ [38]. We note that these bands are related to the MPy-AgNPs SERS bands at 1580, 1588, and 1617 cm^−1^ respectively, and that the mismatch in other band positions are typically 4–7 cm^−1^ (Figure 3a–c). These positional differences can be attributed to a combination of different plasmonic substrate, solution environment, and instrument calibration. In contrast to their results, the inclusion of BSA in our BSA-modified MPy-AgNP resulted in the blue-shifting of the 1580 cm^−1^ band towards that at 1588 cm^−1^ (instead of a red-shift from 1574 to 1582 cm^−1^). Additionally, the 1617 cm^−1^ band was more prominent (Figure 3c). Importantly, the 1588 and 1617 cm^−1^ bands were effectively used for pH sensing. As pH increased, the 1588 cm^−1^ band increased relative to that at 1617 cm^−1^. Multiple interactions are expected to affect these peaks. For example, double-bonded 4-MPy results in a red-shifting the 1610 cm^−1^ band towards 1575 cm^−1^ and can be initiated by laser [45]. Generally, although not necessarily always, red-shifting of the bands near 1617 cm^−1^ to lower wavenumber indicates 4-MPy interactions with species of larger mass and is associated with slower C=C vibration. For example, the shift from 1617 to 1588 cm^−1^ indicates protonation and shifts to lower wavenumbers may be attributed to 4-MPy interactions with other molecules, AgNPs, or ions. The solution environment determines the 4-MPy orientation on the AgNP surface, which affects the SERS spectrum [7,41]. Even hydrogen-bonding contributes to the 4-MPy SERS spectra, implying many potential interferents to the pH sensitive bands [60].

In Figure 3a–c the SERS spectra of MPy-AgNP and BSA-modified MPy-AgNPs in 1× PBS buffer at pH 6–10 are compared. Visual color changes are shown in Figure S4. The MPy-AgNP SERS spectra obtained in buffer nearly match those reported for MPy-AgNP in deionized water, with discrepancies attributed to the different solution environment. The bands at 1617, 1588, 1226, 1099, 1066, and 1012 cm^−1^ are attributed to the deprotonated ring C=C stretching, protonated C=C ring-stretching, C-H in-plane bending, ring breathing/C-S, C-H bending, and ring breathing modes, respectively [41,44]. BSA-modified MPy-AgNP SERS spectra were similar to those of MPy-AgNP, with some differences: the bands 1026, 1203, 1588, and 1617 cm^−1^ became more prominent whereas those at 1281, 1499, and 1580 cm^−1^ decreased or disappeared. The bands at 1026, 1203, and 1617 cm^−1^ changed more with respect to pH in the SERS of BSA-modified MPy-AgNP compared to the SERS of MPy-AgNP, suggesting unhindered 4-MPy protonation and deprotonation (Figure 3c and Figure S5).

A pH calibration curve was constructed for BSA-modified MPy-AgNPs using the intensity ratio of the bands at 1588 cm^−1^ and 1617 cm^−1^, or I@1588/I@1617 (Figure 3d). The band at 1588 cm^−1^ was chosen due to its dominance in both the SERS spectra of MPy-AgNP and BSA-modified MPy-AgNP. A similar plot was constructed with the ratio I@1580/I@1617 (Figure S6). BSA-modified MPy-AgNP had a larger sensitivity (2.6 units/pH) compared to that of MPy-AgNP (0.8 units/pH). The lower (LLOD) and upper limit of detection (ULOD) for the pH calibration of BSA-modified MPy-AgNP were 6.5 and 9.7, respectively (calculated as three standard deviations above and below average signal at minimum and maximum measurements, respectively). For MPy-AgNP the limits are 6.2 and 8.4, respectively. The higher ULOD of BSA-modified MPy-AgNP suggested it was a superior pH sensor compared to MPy-AgNP in the basic region. The lower sensitivity and lower ULOD of MPy-AgNP results from 4-MPy existing in two states. This is evidenced by the comparable intensities of the 1580 and 1588 cm^−1^ bands which decreases the I@1588/I@1617 ratio numerator.

### 3.3. BSA-Modified MPy-AgNP Sensitivity Loss Due to Ca^2+^ Ions

**Figure 4:**
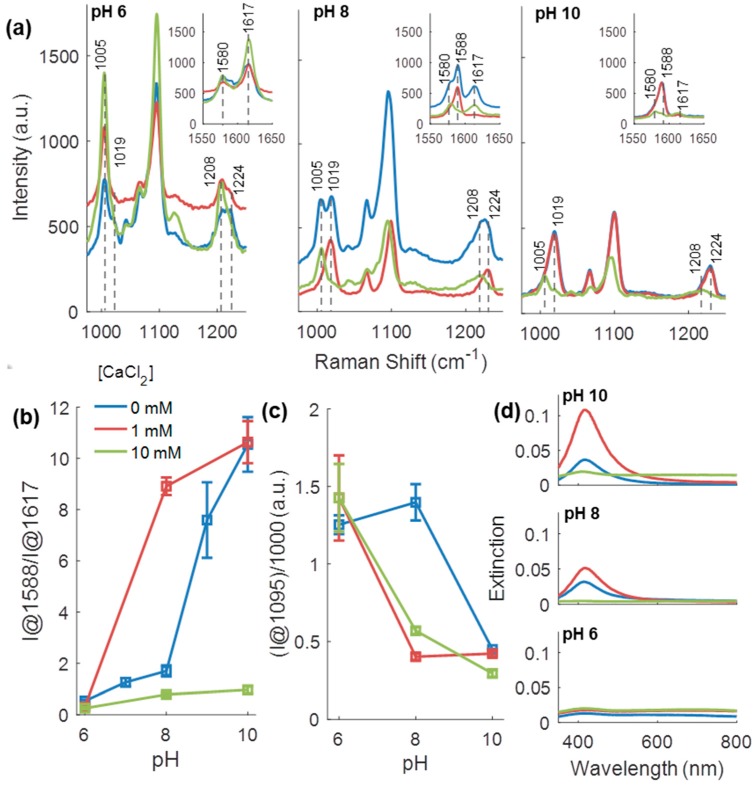
Measurements of BSA-modified MPy-AgNPs in 10 mM Tris-maleate with 0, 1, and 10 mM CaCl_2_. (**a**) SERS spectra without baseline correction or normalization. Inset focused on pH-sensitive bands. (**b**) pH calibration curves calculated from baseline-corrected Raman scattering spectra. (**c**) Intensity at 1095 cm^−1^ band used to gauge scattering amplification. (**d**) UV-vis data. CaCl_2_ concentrations in all plots indicated by color-legend in (**b**). Error bars are one standard deviation (N = 3).

Because alginate hydrogels were stored in buffer containing CaCl_2_ to remain crosslinked, the effect of CaCl_2_ on BSA-modified MPy-AgNP pH response was studied. This study was done in Tris-maleate buffer for comparison to another urease-microcapsule study [8]. The buffer alone affected the pH response. BSA-modified MPy-AgNP in Tris-maleate with no CaCl_2_ (Figure 4b) had one pH response region above and another below pH 8. In contrast, BSA-modified MPy-AgNP in PBS 1× smoothly increased in sensitivity from pH 6–10 (Figure 3d). The SERS bands also differed. The pH-dependent splitting behavior and the location of the bands near 1012 and 1203 cm^−1^ are different for the sample in 1× PBS (Figure 3b) and Tris Maleate (blue lines, Figure 4a). The two buffers have different ionic strength, which may affect 4-MPy deprotonation and protonation.

CaCl_2_ concentration affected the SERS spectra of BSA-modified MPy-AgNP (Figure 4a). At pH 8 and 10 the effect of Ca^2+^ was most evident: a shift between the 1005 and 1019 cm^−1^ bands and shifting between the three 1580, 1588, and 1617 cm^−1^ bands. In addition, at pH 6, the band at 1617 cm^−1^ slightly increased while the band at 1224 cm^−1^ shifted to 1208 cm^−1^ with increasing CaCl_2_ concentration. Other slight differences can be noted. Chloride ions unlikely affected sensitivity: BSA-modified MPy-AgNP was pH sensitive in 1× PBS which contains 137 mM NaCl. Thus, the differences in behavior are attributed to the presence of Ca^2+^ ions.

CaCl_2_ concentration had non-monotonic relationships with the pH sensitive ratios and aggregation state. Aggregation was quantified by two measures: the intensity of the SERS band at the ring-breathing/C-S mode (1099 cm^−1^) and UV-vis (Figure 4c,d respectively). The SERS signal was expected to increase with aggregation, due to more SERS-amplifying hot spots [33]. Aggregation of spherical metal colloids is closely related to surface plasmon resonance peak broadening [61,62,63,64].

In the absence of CaCl_2_ little aggregation was observed at pH 10 as suggested by the narrow UV-vis spectrum (Figure 4d). At pH 8, the extinction peak slightly broadened (Figure 4d) and the intensity of the SERS spectra increased (Figure 4a,c), suggesting BSA-modified MPy-AgNP aggregated. At pH 6, the UV-Vis suggested severe aggregation, although the SERS intensity did not increase compared to the sample in pH 8 buffer.

At 1 mM CaCl_2_ pH sensitivity was lost above pH 8 (Figure 4b) but improved between pH 6–8. Interestingly, at pH 8 a band at 1019 cm^−1^ was found whereas in the absence of Ca^2+^ the band was split into 1019 cm^−1^ and 1005 cm^−1^ (Figure 4a). Furthermore, in the presence of 10 mM CaCl_2_ only the band at 1005 cm^−1^ was observed. The lack of a monotonic relationship between CaCl_2_ concentration and the intensity of the related 1019 cm^−1^ and 1005 cm^−1^ bands may suggest two different mechanisms which affect the 4-MPy behavior in different regimes of Ca^2+^ concentration. 

At 10 mM CaCl_2_ nearly all sensitivity was lost in the studied pH region. The relative increase in the I@1580/I@1617 ratio expected with double-bonded 4-MPy was observed at pH 8 with 1 mM but not 10 mM Ca^2+^ (Figure S7) [45]. In addition, the band at 1617 cm^−1^ generally increased at 10 mM Ca^2+^ suggesting the deprotonated form of 4-MPy might be stabilized at higher Ca^2+^ concentration. Although UV-Vis indicated aggregation in buffer of pH 10 with 10 mM CaCl_2_, the SERS intensity at those conditions was the lowest (Figure 4a,c). This can be explained by BSA-modified MPy-AgNP instability at high Ca^2+^ concentration: their precipitation significantly reduced the dispersion concentration. BSA was shown to bind with increasing quantities of Ca^2+^ as pH increases between pH 7–10 [65], potentially affecting the AgNP-BSA interaction which originally protected the AgNPs. Additionally, AgNP loses colloidal stability due to Ca^2+^ leading to aggregation, which may partially help explain the loss of pH sensitivity [66]. No literature on calcium-MPy interactions were found, although Ca^2+^ interactions with MPy cannot be excluded without further studies.

We highlight the non-monotonic changes in the SERS band intensities with increasing Ca^2+^ concentration implies multiple effects interfere with the BSA-modified MPy-AgNPs pH response. The nature of protonation and deprotonation may be affected by Ca^2+^ interactions with AgNPs, MPy, and BSA, and ionic strength. Further studies are needed to elucidate these effects.

### 3.4. pH Sensing Hydrogel pH Response

**Figure 5:**
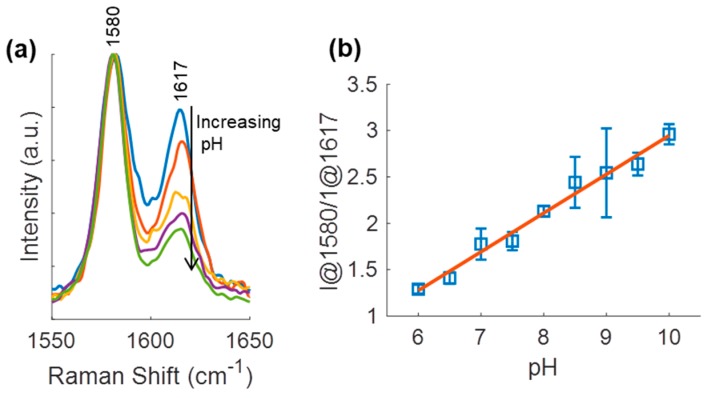
(**a**) SERS pH-sensitive bands for pH sensing hydrogel from pH 6–10 in increments of 1. (**b**) pH sensing hydrogel pH calibration curve. Error bars are one standard deviation (N = 3 discs per pH).

Calibration curves for response to pH were calculated for the pH sensing hydrogel in 10 mM Tris-maleate solution containing 10 mM CaCl_2_ using the I@1580/I@1617 ratio because of the lack of band at 1588 cm^−1^ (Figure 5). The dominance of the 1580 cm^−1^ band may suggest double-bonded 4-MPy or alternative interactions. The pH sensing hydrogel SERS intensity ratio increased linearly with pH, unlike the BSA-modified MPy-AgNP in the same buffer (Figure S7). The pH sensing hydrogel remained sensitive (0.4 units/pH) from pH 6–10 whereas the BSA-modified MPy-AgNP lost sensitivity above pH 8. Between pH 6–8, though, the pH sensing hydrogel had a similar sensitivity to the BSA-modified MPy-AgNP (0.4 versus 0.5 units/pH). The slightly lower sensitivity of the pH sensing hydrogel may be due to alginate contributions to SERS background.

Given the results relayed in Figure 4 and Figure S7, it was surprising that sensitivity was retained in the pH sensing hydrogel while the encapsulated BSA-modified MPy-AgNPs were exposed to Ca^2+^. One possible explanation is that pH-dependent Ca^2+^ interactions between BSA-modified MPy-AgNPs are physically limited by the microcapsule shell. Another potential benefit is the fixed aggregation state within each microcapsule. Assuming a homogenous distribution of AgNPs per microcapsule, each microcapsule contained ~500 AgNPs, 6.4 × 10^6^ 4-MPy molecules, and 7.7 × 10^6^ BSA molecules. Likely, the encapsulation efficiency was not 100%, and some BSA-modified MPy-AgNPs washed away during fabrication [47]. Aggregates containing much greater than 500 AgNPs may be expected with non-encapsulated BSA-modified MPy-AgNP exposed to Ca^2+^, although the only limited evidence of large-scale aggregation provided here is the UV-Vis data in Figure 4. The effect of a Donnan equilibrium on pH sensitivity shift was also considered to be a possible confounding factor, although the high osmolyte concentration was expected to suppress the Donnan effect [49]. 

### 3.5. Urea Sensing Hydrogel Urea Response

To demonstrate direct applicability of the pH sensor to monitor urea, a urea-responsive hydrogel was fabricated by embedding urease within the hydrogel matrix of the pH sensing hydrogel, outside the microcapsules. In the presence of urea, this material design results in urease hydrolysis of urea. An increasing concentration of urea was expected to increase pH throughout the hydrogel and alter the 4-MPy SERS response. Additionally, urea diffusion/reaction and hydroxide diffusion characteristics were expected to yield a time-dependent response of the SERS spectrum [8], where in this case, the measured ratio increases with time in the presence of urea. Similar to the pH sensing hydrogel, the band at 1588 cm^−1^ was not dominant. Thus, the temporal evolution of I@1580/I@1617 ratio was observed to assess this transient behavior. Twelve urea sensing hydrogel discs were split into four groups of three discs and each group immersed in buffer of different urea concentration (10 mM Tris-maleate buffer at pH 7 with 10 mM CaCl_2_ and 0, 0.1, 1, or 10 mM urea). SERS pH measurements were acquired 0, 30, and 60 min after immersion. 

**Figure 6:**
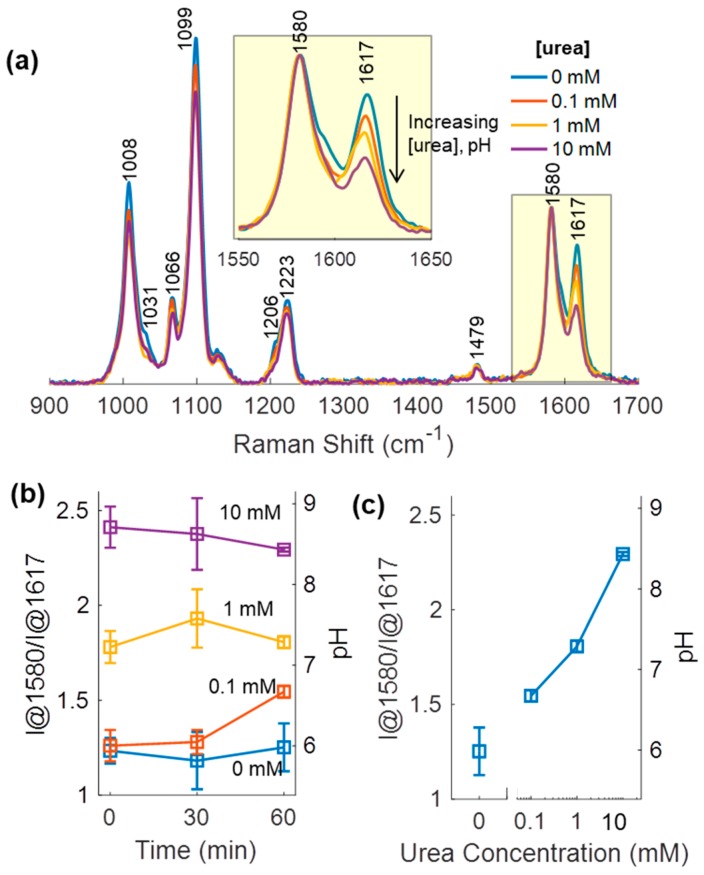
(**a**) Baseline-corrected SERS spectra of urea sensing hydrogel immersed in buffer (10 mM Tris-maleate buffer originally at pH 7 with 10 mM CaCl_2_) containing urea concentrations 0, 0.1, 1, 10 mM, 60 min after immersion. (**b**) Time dependent ratio response for urea sensing hydrogel for 0, 0.1, 1, and 10 mM urea. (**c**) Urea calibration curve corresponding to SERS spectra in (**a**).

Spectral data from these experiments and the corresponding ratio calculations are provided in Figure 6. The intensity ratio of the discs immersed in 0 mM urea was relatively unchanged indicating a constant pH. The buffer pH was 7, although a pH of 6.2 was expected based on comparison of the peak intensity ratio to the pH sensing hydrogel calibration curve in Figure 5. Sample preparation differences may account for the differences between the pH baselines of the pH sensing and urea sensing hydrogels: the two sets of materials were prepared using different batches of BSA-modified AgNPs, which were made from different batches of base AgNPs. Because the 0 mM urea measurement was pH 7 buffer, the pH values in Figure 6 are expected to be lower than the actual values in the microcapsule environment.

Regardless of the baseline shift, urea sensing capabilities were clearly demonstrated using the enzyme-functionalized hydrogels. After immersion into urea-containing buffer, the I@1580/I@1617 ratio decreased indicating a local pH increase resulting from urea hydrolysis (Figure 6a). Immediately after immersion, the intensity ratios of discs immersed in 1 mM and 10 mM urea were noticeably higher than those immersed in 0.1 and 0 mM urea (Figure 6b). This suggested that in the 3 min between sample immersion and the end of Raman data collection, sufficient hydroxide produced by urea hydrolysis had diffused into the microcapsules to alter the local pH. The quick dynamics of this system compared to that of Kazakova’s microcapsule system may be attributed to the higher urease content (9.1 mg/mL versus 1 mg/mL) and the enzyme positioning outside of the microcapsules, meaning greater accessibility to urea [8]. Over time, the peak ratio of urea sensing hydrogel discs immersed in 0.1 mM urea also increased, indicating gradual urea consumption. The constant pH and peak intensity at 1 and 10 mM urea indicate a saturated or reaction-diffusion balanced system. Urease activity decreases at basic conditions as much as 40%, also potentially explaining the lack of further increase of the intensity ratio at 1 and 10 mM urea [67]. 

A representative urea calibration curve for the response after 60 min of urea exposure is shown in Figure 6c (corresponds to Figure 6a and time point of 60 min in Figure 6b). Greater sensitivity was noted between urea concentration of 0–1 mM (0.6 units/mM urea) than between 1–10 mM (0.06 units/mM urea), potentially suggesting a limit of detection between 1–10 mM urea. Tuning the sensor sensitivity to physiological range of 1.8–7.1 mM urea could thus involve lowering the urease concentration. The corresponding pH, predicted by using calibration from Figure 5, is also noted, indicating the very high transduction sensitivity of the system as tested.

## 4. Conclusions

Urea and pH sensors that functioned in the buffered solution were demonstrated. The pH sensor consisted of encapsulated SERS pH reporters (BSA-modified MPy-AgNPs), which were immobilized in alginate hydrogel. This sensor reported between pH 6.5–9.7. The urea sensor consisted of the same encapsulated pH sensor and urease enzyme in an alginate hydrogel and reported urea concentrations of 0, 0.1, 1, and 10 mM. The encapsulation and immobilization of the SERS pH sensor opens up the possibility of SERS-based sensors that measure base-producing enzymatic reactions. By replacing the enzyme in the hydrogel system, and adjusting the enzyme and microcapsule concentrations, new target analytes can be selected for sensing.

Retention of the 4-MPy ligand pH sensitivity in BSA-modified MPy-AgNP was crucial to the success of this urea sensor. BSA protein made this possible. Compared to MPy-AgNP, the pH sensor with BSA (BSA-modified MPy-AgNP) had an extended pH sensing range in the basic region, greater reproducibility, and higher sensitivity, a finding that was attributed to BSA inhibiting aggregation and thus preventing undesired cross-nanoparticle interactions. Our findings further suggest that BSA protected BSA-modified MPy-AgNP from excessive aggregation during CaCO_3_ microspheres co-precipitation. While Ca^2+^ ions directly impacted the pH sensitivity of BSA-modified MPy-AgNP, the encapsulated form of BSA-modified MPy-AgNP was prevented further aggregation in the presence of Ca^2+^. It is noteworthy that low-cost proteins, such as BSA, can limit the negative effect of the encapsulation process on SERS-based pH sensors that are otherwise prone to aggregation. BSA could be seen as an effective encapsulation enhancer in this case. Further studies are needed to more completely characterize the role of microcapsules in restricting aggregation, and the mechanisms that affect 4-MPy response to pH due to aggregation.

## Supplementary Materials

The following are available online at https://www.mdpi.com/1424-8220/19/16/3521/s1, Figure S1: Photographs of AgNPs, MPy-AgNPs, and BSA-modified MPy-AgNPs, Figure S2: CaCO_3_ microspheres containing BSA-modified MPy-AgNPs, Figure S3: Urea sensing hydrogel, Figure S4: Photographs of BSA-modified MPy-AgNP and MPy-AgNP at different pH, Figure S5: Comparison of pH-sensitive SERS bands for MPy-AgNP and BSA-modified MPy-AgNP, Figure S6: pH calibration curve using I@1580/I@1617 ratio, Figure S7: pH calibration curve using the I@1580/I@1617 ratio for BSA-modified MPy-AgNP in 10 mM Tris Maleate Buffer containing 0, 1, and 10 mM CaCl2.
