# Supplementary material for: Hydrogel Microdomain Encapsulation of Stable Functionalized Silver Nanoparticles for SERS pH and Urea Sensing
Source: Sensors (Basel). 2019 Aug 12;19(16):3521. doi: 10.3390/s19163521 (PMC6720209; doi:10.3390/s19163521)
Supplement: Supplementary file 1 [file sensors-19-03521-s001.pdf]

Supplemental Information

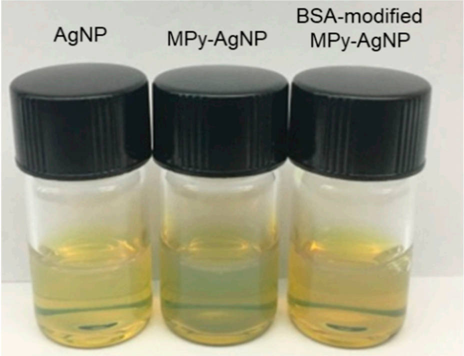

Figure S1 Photographs of AgNPs, MPy-AgNPs, and BSA-modified MPy-AgNPs (left to right, respectively). From lightest to darkest are AgNPs, BSA-modified MPy-AgNPs, and MPy-AgNPs.

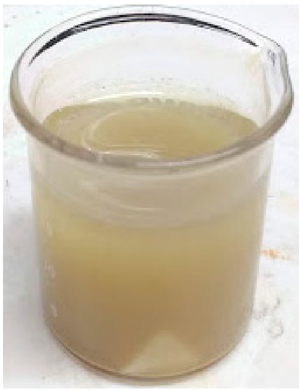

Figure S2 $CaCO_3$ microspheres containing BSA-modified MPy-AgNPs. In absence of BSA (microspheres containing MPy-AgNPs) a dark gray color results instead of the yellow-gray color seen above.

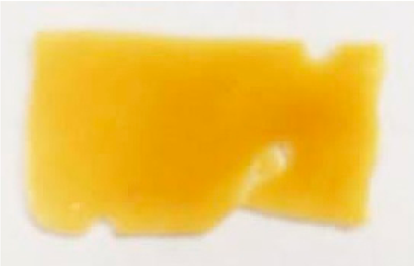

Figure S3 Urea sensing hydrogel which contains microcapsules with BSA-modified MPy-AgNP cargo and urease conjugated to the matrix. The yellow color results from the BSA-modified MPy-AgNPs.

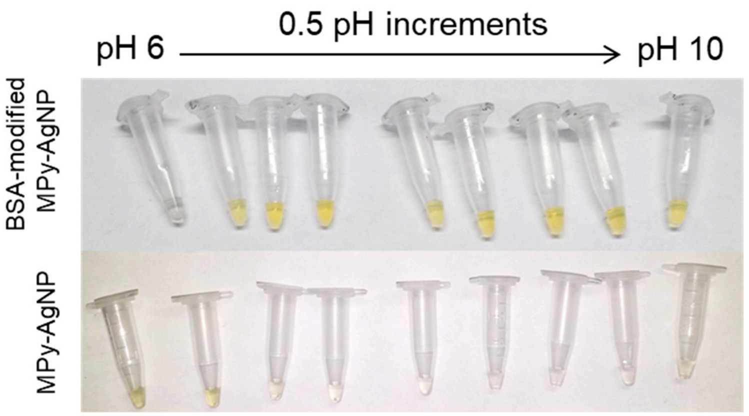

Figure S4 Photographs of BSA-modified MPy-AgNP and MPy-AgNP at different pH. BSA-modified MPy-AgNP retains yellow color over wide pH range, indicating colloidal stability whereas MPy-AgNP only exhibits yellow at lower pHs.

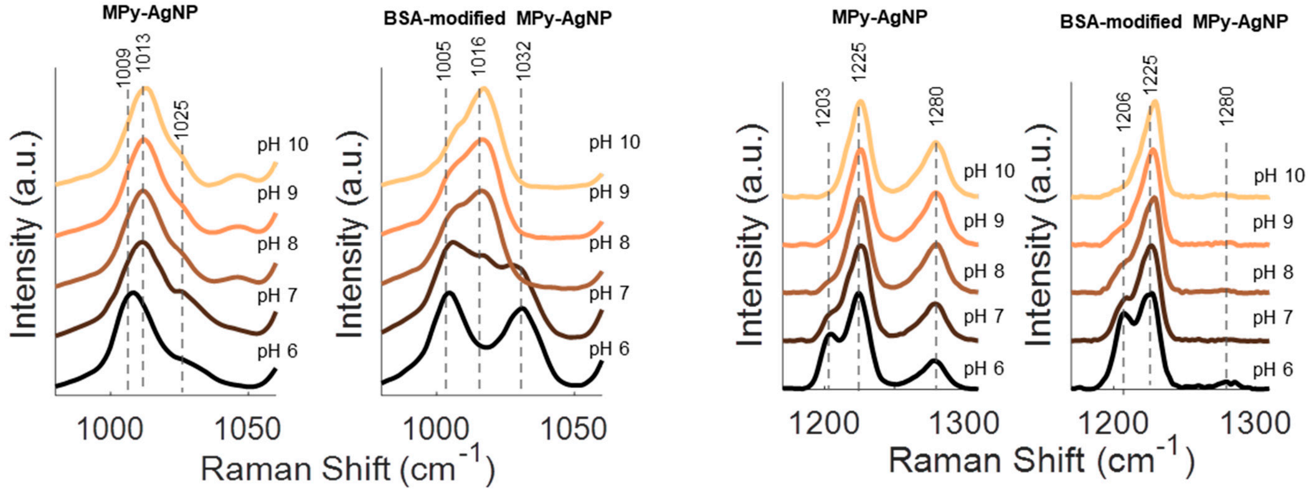

Figure S5 Comparison of pH-sensitive SERS bands for MPy-AgNP and BSA-modified MPy-AgNP.

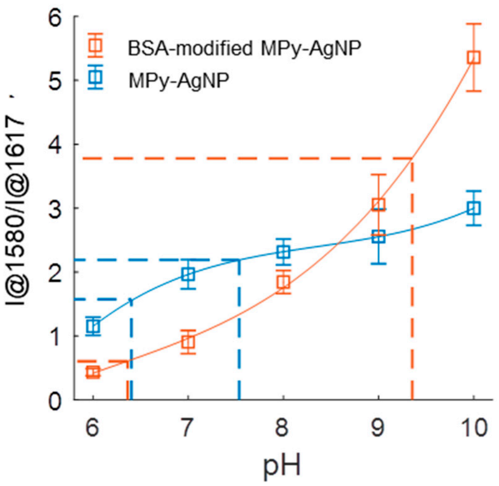

Figure S6 pH calibration curve using I@1580/I@1617 ratio. The band at 1580 cm^{-1} disappears for BSA-modified MPy-AgNP. LLOD and ULOD for MPy-AgNP using I@1580/I@1617 are 6.4 and 7.5 units pH respectively. The sensitivity of MPy-AgNP is 0.5 units/pH. LLOD and ULOD for BSA-modified MPy-AgNP are 6.4 and 9.4 units pH respectively. The sensitivity of BSA-modified MPy-AgNP is 1.2 units/pH.

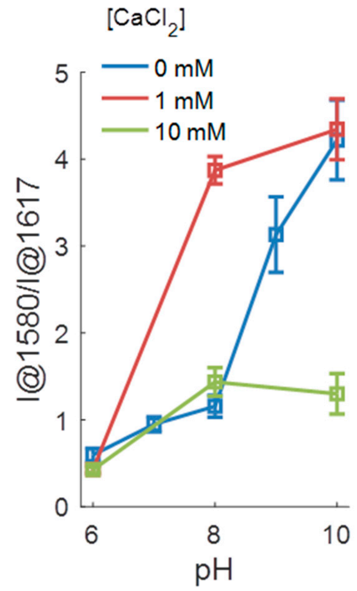

Figure S7 pH calibration curve using the I@1580/I@1617 ratio for BSA-modified MPy-AgNP in 10 mM Tris Maleate Buffer containing 0, 1, and 10 mM $CaCl_2$.
